# Biodegradable Materials and Metallic Implants—A Review

**DOI:** 10.3390/jfb8040044

**Published:** 2017-09-26

**Authors:** Mythili Prakasam, Janis Locs, Kristine Salma-Ancane, Dagnija Loca, Alain Largeteau, Liga Berzina-Cimdina

**Affiliations:** 1 CNRS, Univ. Bordeaux, ICMCB, UPR 9048, F-33600 Pessac, France; alain.largeteau@icmcb.cnrs.fr; 2 Rudolfs Cimdins Riga Biomaterials Innovations and Development Centre of RTU, Institute of General Chemical Engineering, Faculty of Materials Science and Applied Chemistry, Riga Technical University, Pulka 3, LV-1007 Riga, Latvia; janis.locs@rtu.lv (J.L.); kristine.salma-ancane@rtu.lv (K.S.-A.); dagnija.loca@rtu.lv (D.L.); liga@ktf.rtu.lv (L.B.-C.)

**Keywords:** bioceramics, biocomposites, biodegradable metal alloys, bioimplants

## Abstract

Recent progress made in biomaterials and their clinical applications is well known. In the last five decades, great advances have been made in the field of biomaterials, including ceramics, glasses, polymers, composites, glass-ceramics and metal alloys. A variety of bioimplants are currently used in either one of the aforesaid forms. Some of these materials are designed to degrade or to be resorbed inside the body rather than removing the implant after its function is served. Many properties such as mechanical properties, non-toxicity, surface modification, degradation rate, biocompatibility, and corrosion rate and scaffold design are taken into consideration. The current review focuses on state-of-the-art biodegradable bioceramics, polymers, metal alloys and a few implants that employ bioresorbable/biodegradable materials. The essential functions, properties and their critical factors are discussed in detail, in addition to their challenges to be overcome.

## 1. Introduction

Significant advances in science and technology have contributed to various domains, including the medical field. Advances in the field of medical sciences and engineering have created the possibility to employ implants in the human body. Research and development as early as the 1890s, led to the development of pacemakers and defibrillators [1]. Implants appeasing the requirements for replacing/functioning/assisting the natural organs in the human body are called bioimplants. Biocompatibility and implants’ various roles to match the natural bodily functions are studied. Bioimplants [2] in today’s medical scenario are used as brain/neural implants [3], sensory implants [4], spinal implants [5], organ stimulation implants [6], subcutaneous implants [7], dental implants [8], cosmetic implants [9], convenience implants and other structural implants [10], such as stents, braces, rods, heart valves, bones, pins, hip prosthesis, eye, ear, skull implants and knee replacement implants. An upsurge in bioimplants markets is predicted to reach US$116 billion by 2020, primarily because of the rise in road accidents and trauma injuries. Similar demands are also analogous in the domain of cardiovascular, spinal and ophthalmic disorders, leading to a rise in the demand for bioimplants.

Bioimplants are protheses made for regularizing physiological functions, and bioceramics represent one classification of bioimplants based on material type. The classification of biomaterials [11] includes single crystals, polycrystals, glass, glass-ceramics, polymers and composites. The type of interaction and the mechanism of tissue attachment at the implant interface is based on its inherent nature. The type of tissue implant is considered (1) biotoxic if the surrounding tissue dies; (2) it is bioinert-nontoxic and biologically inactive; (3) it is bioactive-nontoxic and biologically active; and (4) bioresorbable/biodegradable-nontoxic and dissolves itself to replace the surrounding tissues. Forms and shapes of bioceramics [12] are available as dense, porous, bimodal and amorphous states. To avoid the complications of ceramics which are bioinert, the bioactive coating of hydroxyapatite (HAp) is used. Ceramic implants and resorbable/biodegradable bioceramics are the focus of this review. Resorbable bioceramics [13] are designed to gradually degrade in a time frame followed by a replacement of natural host tissue. Some of the complications related to resorbable bioceramics are maintaining strength and stability for its whole lifetime (until complete degradation, rate of resorption and the compositional changes in the body). Ideal bioactive [14] ceramics should be biocompatible, osteoconductive, osteoinductive, enable angiogenesis, biodegradable, nontoxic and have the flexibility to form different shapes. Bioceramics based on calcium phosphates (CP) with the mineral component similar to natural bone are a very well-known class of materials used for tissue engineering applications such as scaffolds.

Reconstructive surgery using interference screw fixations is prevalently employed in the femur and tibia [15]. Prior to this, bioresorbable implants and metallic biomaterials [16] were used. The disadvantages of metallic biomaterials are varied; for example, the requirement of postoperative complications, distortion of post-operative metallic screw and inflammatory reactions. Recent progress in development of bioresorbable implants for traumatology and sports injuries [17] is continuously evolving. However, there are still reports about the disadvantages of bioresorbable biomaterials, such as inflammation, osteolysis and the decrease in mechanical strength. Polymers [18] are used as bioresorbable materials for clinical applications for bone implants, sutures, and as drug delivery devices.

Biodegradable materials, irrespective of their constituent form, are expected to degrade progressively over a period of time to assist as scaffolds or for the healing process. Metals are stiff and have high mechanical strength in comparison to their polymer counterparts; hence, the former is usually chosen for medical applications. Magnesium [19] and iron [20] based biodegradable alloys are expected to degrade with the metabolism at an appropriate degradation rate inside the human body. Porous Mg alloy degrades rapidly in vivo. The main characteristics that are considered for porous metallic implants are porosity, pore size and pore interconnectivity, to cater for the replication of natural human-like implants. Higher porosity aids in angiogenesis. Bone is comprised of two distinct forms: one is porous (cancellous bone) and the other dense (cortical bone) [21]. Cancellous bone contains hemocytoblasts, proerythroblasts and bone marrow. Cancellous bone has a lower Youngs modulus and is more elastic compared to cortical bone. The porous structure consists of pore sizes in the range of 200–500 μm, and cancellous bone constitutes 30–90% of the porosity. The porosity content alters depending on the load, age and health state of the bone. Cortical bone is the outer layer of the bone that aids in providing the shape and form of the bone. Eighty percent of the skeleton is composed of cortical bone. Cortical bone stacks osteons or Harversian systems in the form of interstitial lamellae. In the case of loss of bone, bone grafting is used, and solutions are chosen based on the required biomechanical properties, chemical composition, bone mass and size of the defect site. Different types of bone grafting methods are employed [22]. A few of the bone grafting methods are autografting (cancellous/cortical bones), allograft (cancellous/demineralized bone matrix (DBM)) and bone graft substitutes (HAp/tricalcium phosphates Ca_3_(PO_4_)_2_(TCP)/biphasic calcium phosphates (BCP)/bioactive composites, growth factors).

The objective of this paper is to review the progress and development of biodegradable/bioresorbable biomaterials for various applications and their current challenges and future developments.

## 2. Bioresorbable Composites and Implants

### 2.1. Bioceramics

Scaffold design factors [23] in bone tissue engineering are given in Table 1. Biomaterials are considered as the alternative solution for natural bone with similar osteoinduction, osteoconduction, inflammation and mechanical integrity as native bone. Usage of bioceramics implants avoids the risk of disease transmission and immunogenicity. Bioceramics will experience humid conditions upon implantation. Currently, bioceramics are used for applications [24] related to dental, periodontal, cochlear, maxillofacial, spinal discs and otolaryngology. Initially, developed bioinert bioceramics were principally from zirconia (ZrO_2_) [25] and alumina (Al_2_O_3_) [26] to fabricate femoral heads of total hip arthroplasty prostheses. The aforesaid ceramics are well known for their good mechanical properties. Later, bioactive ceramics were developed and are currently widely known for their ability to form the bone bonding without causing inflammation. Calcium phosphates (CPs) [27] have high biocompatibility which led to studies on a variety of CPs. Some of the CPs based on hydroxyapatite (HAp, Ca_10_(PO_4_)_6_(OH)_2_), dicalcium phosphate dihydrate (DCPD, brushite, CaHPO_4_·2H_2_O) and tricalcium phosphate (TCP, Ca_3_(PO_4_)_2_) are extensively studied as bone replacement materials. β-TCP exhibits higher resorbability than HAp under physiological conditions. Bioceramics are commonly used for various applications such as fracture repair, bone defect filling and as bone replacement tissues. Degradable bioceramics [28] (Table 2) have a significantly lower mechanical strength in comparison to their non-resorbable counterparts. The physical and chemical composition of bioceramics determines their biological response. Bioactive ceramics also assist in the production of proteins and cell adhesion (osteoconduction) because of their presence of inorganic phase (CP). Bioceramics, with their capability of stimulating bone regeneration tissues, have led to a new class of biomaterials.

Tricalcium phosphate (TCP) [29] is known to have a close chemical composition similar to bone tissue mineral. TCP has two different crystalline forms: α-TCP and β-TCP. TCP has a good resorbability and bioactivity with a higher rate of biodegradation than hydroxyapatite under in vivo conditions [30]. Due to its osteoconductivity and resorption properties, it is widely used for bone graft remodeling. TCP is also used for the applications in orthopedic, dental and maxillofacial applications. Complete resorption of TCP is reported in the case of rat tibia and for cancellous bone reformation in canine models. Bone grafts made of TCP show a slow resorbability by osteoclasts over a span of 10 months to 2 years.

Calcium phosphate is the major inorganic component of bone tissue, with hydroxyapatite (HAp) having an atomic ratio of 1.67 for calcium and phosphate. HAp is bioactive and resorbable (when it is either in amorphous or low crystalline state). Natural and synthetic HAp reactivity varies based on the preparation method. Synthetic HAp prepared at high temperature yields high crystallinity. Biodegradation and resorbability of HAp are very slow. HAp bioceramics are commonly used for the small defects in the case of bone loss or in the case of fractures of the tibia, calcaneus and vertebra. HAp is not employed for load-bearing bone applications because of the poor mechanical properties. Currently, there is huge progress being made towards the development of HAp with improved mechanical and biological properties for tissue engineering. Metal ions such as strontium, magnesium and silicon are substituted in HAp, and have resulted in the improvement of biological and mechanical properties [31]. Zinc- [32] and manganese- [33] doped HAp has a quick resorption mechanism. Continuous research on the improvement of properties of HAp has shown a good response in terms of osteoconductivity and osteogenicity with future prospects to be employed as a bioactive delivery vehicle.

Dicalcium phosphates (DCP) [34] consist mainly of calcium phosphates and water, and are commonly used to alter the physical properties of the composition. A dehydrated form of DCP is known by the mineral name brushite, with the Ca/P ratio 1. DCP-based bioceramics are biodegradable. Brushite is known to convert into HAp in vivo or under physiological conditions that restricts its resorbability and degradation rate. Brushite is used for the treatment of fractures in tibial plates, distal metaphysis bone. Monetite (CaHPO_4_) bioceramics [35] are also reported for their applications in bone augmentation and regeneration for dental and orthopedic applications. The bioceramics [36,37,38,39,40,41] which are employed for the applications in hard tissue replacement are zirconia and alumina (and are also commonly employed in the fabrication of femoral heads).

### 2.2. Biodegradable Polymers

Natural-based [42] and synthetic based [43] polymer materials are some of the preferred choices for biodegradable materials. Polymers can be defined as macromolecules composed of covalently bonded monomers. Depending on the number of repeating monomers belonging to the same or different molecules, they can be classified as homopolymers and copolymers, respectively. Some of the known nature-based polymers are starch, chitosan, derivatives of hyaluronic acid, collagen, fibrin gels and silk. Practical applications of the aforesaid natural-based polymers are impeded by their low mechanical strength, unknown rate of degradation, repellency and high physiological activity. To overcome the challenges posed by natural polymers and in order to replicate them, synthetic polymers (Table 3) were studied with catered design of properties according to the requirements. Depending on the type of polymer chain arrangement—linear, branched, or cross-linked [44]—and its crystallinity/amorphous nature, the degradation rate can be altered. The glass transition temperature of polymers makes them flexible [45]; hence, it is necessary to make polymers biodegradable above normal body temperature. Some of the commonly used biodegradable polymers [46] are polyglycolic acid (PGA), polylactic acid (PLA), poly-β-hydroxybutyrate (PHB), poly (lactic acid-*co*-glycolic acid) (PLGA) and poly-ε-caprolactone (PCL). Among these polymers, PLA, PGA and PLGA are used for degradable sutures. PLA was the earliest reported polymer used for sutures [47]. PLA was synthesized from natural sources such as starch and corns. Crystalline PLA (L-PLA) is less resistant to hydrolysis than amorphous PLA (DL-PLA) [48]. PGA, a synthetic polymer, has high crystallinity, low solubility and high degradation rate (because of hydrophilism). The high degradation rate of PGA results in the decrease of its mechanical strength after implantation [49]. PCL, an aliphatic polyester, although difficult to control its degradation rate, is widely used in medical implants. PCL is usually employed in the case of long-term implants and as a drug delivery system owing to its crystallinity and permeability, respectively. PCL also has high flexibility and shaping options to form different shapes. PHB is another type of biodegradable polymer with high crystallinity and is used as a model for physical properties of polymers [50]. However, due to the brittleness of PHB, it is not employed for practical applications, but is blended with other polymers to study their miscibility and crystallization properties. Poly-para-dioxanone (PPD) [51] is another biodegradable polymer which is commonly used for its biodegradability, resorbability, compatibility, and its flexibility as a medical implant. PPD is used as fracture internal fixation material and in the form of films, foams, molded products and coatings. Studies have shown the possibility of complete disintegration of PPD implants within 6–7 months after implantation by varying the molecular weight, crystallinity and their melting temperature [52].

The choice of biodegradable polymers is chosen based on the required properties, such as their chemical, physical and mechanical properties. PLA, classified on the basis of its stereoisomers such as poly (l-lactide) acid (PLLA) and poly (d-lactide) acid (PDLA) [53], is influenced by their constituent polymer chains which determine PLA’s characteristics. The degradation of the polymers is through hydrolysis of ester bonds resulting in alteration of their chemical structure. When the surface of the polymer is attracted by the microorganism, the enzymes secreted by the microorganism converted the macromolecules into tiny molecular debris and eventually converted them into CO_2_ and H_2_O. A physical biodegradation process occurs as the microorganisms interact with the surface of the polymers, resulting in hydrolysis (which is a chemical process) to ionize and convert them into oligomer debris. However, chemical biodegradation occurs due to the enzymatic interaction of the microorganism with polymers leading to conversion into CO_2_ and H_2_O. However, it has to be noted that all the biodegradation processes of all the polymers do not follow the same methodology, and the complex process of biodegradation is studied extensively by various research groups [54,55,56].

Implants for bone repair and other load bearing implants require high mechanical strength; hence, ceramics and metals are preferred. However, in some cases, counter-effects such as inflammation [57], corrosion [58] and bone loss [59] are observed. Hence, polymers are considered for bone repair applications [60] owing to their tensile strength, elastic modulus and non-corrosion properties. It has to be noted that polymers also experience a loss of mechanical strength with the increase in implantation time as a result of degradation/resorbability. Currently, polymers such as PGA, PLA and PLGA are widely employed for bone implants. Other fields of applications include screws, bone fixation objects, anklebones, patella and other fixing objects. Some disadvantages of these polymers involve X-ray transparency, unidentified influences of foreign body interactions and decreased mechanical strength with time. Other types of polymers such as collagen, hyaluronan and fibrin glue are also degraded enzymatically through collagenase, hyaluronidase and plasmin, respectively.

Cyanoacrylates [73] are known to be super glues or instant adhesives in surgery. The mechanism of degradation appears to involve base-catalyzed unzipping from the chain terminus, followed by re-equilibrium of the chains. The degradation decreases adhesive bond strength on heating and poor resistance to prolonged contact with water [74]. More intensive research is currently being carried out on polymers.

### 2.3. Magnesium Alloys

From the biomedical point of view, magnesium alloys with appropriate mechanical properties and biodegradation are necessary, as well as their biosafety in terms of toxicity. Magnesium alloys are used for tissue engineering, orthopedic and cardiovascular applications. Nan Li et al. [75] reported on the novel magnesium alloys developed for biomedical applications. Witte et al. [76] investigated in vivo corrosion of different types of magnesium alloys and reported that an accumulation of biological calcium phosphates and all related alloys displayed a better osteointegration. The mechanical properties of magnesium alloys are increased by alloying with aluminium [77] and rare earth elements [78]. However, owing to the neurotoxicity due to the accumulation of aluminium [79] and hepatotoxicity of rare earth materials [80], the advantage of increasing mechanical properties of Magnesium alloys cannot be used for biomedical applications. There is extensive research pointed in this direction to identify the new magnesium alloys with nontoxicity or low toxicity [81]. Various biomedical magnesium alloys such as Mg–Zn-based [82], Mg–Ca-based [83], Mg–Si-based, Mg–Sr-based and Mg–rare earth alloy-based are studied extensively [84] by various research groups for the development of biodegradable magnesium alloys. Rapid corrosion [85] of pure magnesium is well-known, but with the increase of purity by purification, the corrosion rate is reduced to a large extent. The common impurities found in magnesium are Fe, Cu and Ni [86]. The corrosion rate of magnesium is highly dependent on the ratio of impurities present in it. Pure magnesium cannot be an apt material for applications in orthopedics because of their mechanical properties. Coarsening of magnesium grains [87] is caused by their heat treatments such as forging or rolling. Calcium (Ca) is used for grain refining in magnesium alloys [88]. The solubility limit of Ca in Mg matric is only 1.34 wt %. There are also reports on the increase of coarse grains in Mg–Ca alloys in the case of increase in Ca [89]. Rapid solidification [90] of Mg–Ca alloys yielded fine grains that had superior corrosion resistance and improved cell activity. Degradation [91] of Mg–Ca alloys was reported over a period of 3 months following the bone formation. Zinc (Zn) also forms alloys with Mg through which the improvement of mechanical properties of Mg–Zn alloys can be achieved. Other than binary Mg–Zn alloys [92], ternary and quaternary alloys of Mg–Zn with Zr, Y, Mn and Ca are formed [93]. A binary alloy of Mg–6Zn [94] is resistant to galvanic corrosion due to its single phase formation; in vitro cytotoxicity tests indicated that it has good biocompatibility properties; in vivo tests proved that it is not harmful to the internal organs [95]. However, the bio corrosion was slightly higher in the case of in vivo conditions than in the in vitro conditions. The biodegradation of Mg–Zn alloy was rather average with around 2 mm/year while used as alloy rods in rabbit femur shafts; adding Y in the binary alloy of Mg–Zn aids to diminish the corrosion rate. On the other hand, the addition of Zr in the binary alloy of Mg–Zn helps in grain refinement, and at the same time helps to increase the osteointegration properties superior to titanium alloys. Adding the intermetallic group of metals such as manganese (Mn) to Mg–Zn can contribute to the corrosion-resistive properties and subsequently remove heavy metals such as Fe. The degradation of Mg–Zn–Mn alloy is highly dependent on the individual constituent of each component in the alloy. The alloy compound of Mg–Zn–Ca [96,97] has superior mechanical and corrosion properties when Zn content is <4 wt %, even under in vivo conditions. Other alloys of Mg with Zn, Ca and Si and Sr are also prevalent. The aforesaid were found to contribute primarily to the refinement of grains and change of grain morphology, which assists in corrosion resistance. Although rare earth elements were added as dopants, the toxicity of the rare earth elements is well known. Hence, rare earth elements are not yet employed as implant materials. In the current state-of-the-art [98], magnesium-based alloys cannot be used for orthopedic applications due to their high degradation rate. Concerning the corrosion rate, magnesium alloys based on Mg–Mn–Zn and Mg–Ca have a low in vivo corrosion rate. The mechanical strength of these Mg-based alloys is increased through the change in their structural properties because of their biocompatible properties. The fabrication methodology [99] through powder metallurgy, melt-casting and spin melting, is used for the fabrication of magnesium alloys and magnesium-based metal composites. Another strategy employed for controlling the degradation rate of magnesium alloys is through coating their surfaces with calcium phosphate [100], fluorinated coatings, polymer coatings, etc. Magnesium-based implants are currently used as micro clips for laryngeal microsurgery, orthopedic and cardiovascular systems. There are further developments in this domain of developing magnesium alloys. The common challenge in magnesium alloys is their formation of hydrogen. Magnesium glasses are considered as an alternative for crystalline alloys to avoid the formation of the hydrogen bubble effect [101]. During the manufacturing process of magnesium alloys, their influence on the implant surface with controllable properties is required for controlling the degradation and corrosion process.

### 2.4. Titanium Alloys

Titanium, being one of the low-density elements, is one of the materials used for biomedical applications [102]. The strengthening process of a titanium alloy is done either by alloying or deformation processing [103]. Ti alloys were favored as biomaterials because of their good biocompatibility [104], corrosion resistance [105] and lower modulus [106]. Ti alloys such as α + β Ti–6Al–4V and metastable β alloys are currently being investigated [107]. Pure titanium undergoes a transformation temperature from α phase to β phase at ~885 °C. The transition temperature is influenced by the interstitial elements such as, O.; N, C, B and H and various other substantial elements. The microstructure and properties of Ti alloys vary depending on the type of interstitial/substitutional element chosen [108]. Many substitutional metals such as, V.; Mo, Nb, Cr, Fe, Si, Ta, Cu, Ni, Pd, Co, M, and W are used as additives to stabilize the β phase. Titanium alloys have considerably superior biocompatibility compared to their other counterparts due to their excellent corrosion resistance. In comparison to Ti–6Al–4V, β-titanium alloys with stabilizing elements (Mo, Ta and Zr) are considered to be safe. Β–titanium alloys have a modulus closer to that of bone. However, the fatigue strength is poor compared to other α–β alloys. The fatigue strength of these alloys was enhanced by the addition of Y_2_O_3_, SiO_2_ and ZrO_2_ which causes dispersion; hence, strengthening the alloys. Ti alloys require surface modifications to increase their wear resistance. Titanium alloys form easy bonding with bone, demonstrating good integration with bone tissue. Currently, pure Ti alloys are used in pacemaker cases, ventricular devices, implantable drug pumps, screws and staples in spinal surgery, dental implants and in craniofacial implants. Ti–6Al–4V alloys are used for dental implants, hip, knee, wrist, spine, shoulder, elbow and orthodontics. However, there is a long-term risk associated because of the release of Al and V that causes Alzheimer’s, osteomalacia and other neurological conditions. Currently, Vanadium free Ti–6Al–7Nb and Ti–5Al–2.5 Fe are being developed [109] and used in femoral prosthesis stems, fracture plates, nails, rods, screws and wires. Titanium alloys cause allergic tissue reactions because of fretting, and have relatively less wear resistance in comparison to other alloys used. Because of the poor bending properties of α–β titanium alloys, Ti–Mo–Zr–Fe [110] was used instead for total hip replacement prostheses. Titanium alloy’s wear resistance is improved by addition of refractory metal elements and by surface modification techniques [111]. The application of Ti alloys for implant materials is impeded by their poor mechanical strength.

### 2.5. Orthopedic Implants

Orthopedic implants is the term representing implants that replace a missing joint or any damaged bone. Orthopedic implants represent a large part of the implants made in the field of medical implants. Orthopedic implants are mostly composed of chromium, cobalt, molybdenum, nickel, titanium and zirconium alloys. The disadvantages of metallic orthopedic implants such as metal allergy, metal implant failure, and complications related to joint replacement are reported. Other serious complications of orthopedic implants include device failure, pain, metallic staining of the surrounding tissue, muscular necrosis, periprosthetic fibrosis and loosening of prosthesis. Orthopedic implants used for total hip arthroplasty (THA) and total knee arthroplasty (TKA) metal allergy and device failure are reported. Metallic orthopedic implants failure results in pain, instability, malrotation, and local pain leading to infection. Wendy et al. reported on the metal hypersensitivity reactions to orthopedic implants [112]. Other orthopedic implants also include bone screw with self-tapping threads used as fastening elements in prosthetics. Steel and titanium screws are used prevalently for fabrication of bone screws. Recently, there has been progress made in the direction of better performance of bone screws leading to the study of magnesium (Mg) and Mg-coated screws. Recently, Ullrich et al. [113] reported on the bone screw with minimum risk of fracture with miniaturization, by reducing the friction on the surface of the screw implant. This was achieved by means of surface functionalization, self-tapping and self-lubricating thread profile by incorporating some bioactive materials to accelerate bone growth/healing. By improving the tapping efficiency of the abrasives, friction is minimized during the screwing-in process. The mass and size of the implant is reduced which will reduce patient rehabilitation time; this also opens avenues for resorbable implants such as in the skull and small limb field.

### 2.6. Metals Used in Knee Replacement

Many different types of metallic systems are used for knee replacement. Steel has higher stiffness than the cortical bone with an elastic modulus of 200 GPa [114]. Stiff steel implants bare all the load, and thus weaken the normal bone. Hence, bone cement is used as an interface to decrease the stress shielding effect [115]. Fixing of stiff steel implants is usually carried out with the help of bone cement. Most commonly used steels are cheaper than other types of metals. In medical fields, the commonly used iron alloys are of low carbon alloyed, chromium (17–20%), molybdenum (2–4%) and nickel (12–14%), with the addition of other small amounts of other elements. The corrosion resistance of these metals is under intensive investigations [116] as release of these metal ions inside the body could cause allergy, toxicity and other symptoms. The release of iron from stainless steel favors bacteria infections by acting as an iron source for their proliferation [117]. To avoid these inconveniences, stainless steel implants are used in the body as short-term implants or as coated implants. Recently, cobalt- and titanium-based materials were used as joint replacement materials. The fabrication methodology and the microstructural defects lead to fatigue-based fracture. Good fatigue resistance of approximately 400 MPa is considered necessary for total hip implants to last a long time [118]. Fatigue resistance is diminished by wear, corrosion and material defects. Steel has an elastic modulus of approximately 200 GPa and titanium has an elastic modulus of approximately 110 GPa. Hence, while using the titanium-based alloys, cement used as an intermediate layer is not considered important. To ensure fixation, porous-coated implants are used. Hydroxyapatite is commonly used as a coating material. In total joint arthroplasty, aseptic loosening is the prime cause of failure. Stainless steel knee implants were reported to have an 80–90% success rate, even after 25 years of implantation [119]. Radiographic loosening was inferred in few cases of cobalt–chrome alloys. As a cementless implant, the surface finish governs the osseointegration potential.

### 2.7. Orthodontic Wire

Orthodontic brackets and wires are widely used in cases of a long span of treatments. Researchers are investigating new types of archwires to minimize the discomfort caused [120]. Various materials such as nickel titanium, beta titanium, copper and stainless steel are currently used. Different shapes and forms (such as square, round, rectangular and beveled surfaces) of orthodontic archwires [121] are used. There was continuous evolution between the archwire and the bracket to reduce the friction significantly. Heat-activated multimodulus wire, due to its increased flexibility, minimizes angular friction. Nickel titanium (NiTi) wires [122] with added resiliency and varying heat activation variation is considered mostly by dentists, whereas the overstressing of the NiTi wire causes permanent distortion. To reduce the wire surface friction, surface coatings are employed on NiTi wires. Beta titanium wire appears to be the intermediate wire between NiTi wires and stainless steel, with twice the flexibility and half the force of stainless steel. Another material, copper NiTi, is reported to be more resistant to permanent distortion than other nickel titanium wires [123]. Various materials in the following classifications such as noble metal alloys, stainless archwires, cobalt–chromium archwire, nickel titantium archwire, copper nickel titanium alloy and beta titanium archwire are used for archwires. However, detailed information on these materials is not given here as they are out of the scope of the present review article. Biodegradable metals are also used as metallic stents. Biodegradable metals are used for treating coronary artery diseases. Mg, Fe and Zn are widely used for biomedical applications. Mg–6Zn alloys, Zn, Fe and Co–Cr stents are biocompatible, but the latter two have less degradation time.

## 3. Conclusions

Bioresorbable/biodegradable composites and ceramic implants are of growing interest because of their ability to provide a necessary mechanical function for the tissue reconstruction process after the designated function in the body. These bioresorbable materials should degrade at a tolerable range for the body and an optimized degradation rate is required based on the function that the material serves in the body. There are not enough human in vivo data to validate the biodegradability of bioresorbable materials. This inhibits the valorization of the materials for marketing purposes. There are many challenges, such as faster than expected degeneration of the mechanical properties of Mg-based alloys during the tissue remodeling process. Surface coatings and modifications are suggested as an alternative to achieve the extended mechanical integrity of these materials. More research has to be focused on materials such as Mg-based and Fe-based alloys to determine their mechanical and degradation kinetics. The biosafety of the degrading material should be ensured with the host tissue and implant interface. Various other parameters such as age, physical condition, risk of infection, and type of fracture are primordial for the choice of the required bioresorbable composites and ceramic implants. Currently, biodegradable/bioresorbable polymers are successfully used for biomedical and pharmaceutical applications. Polymers such as PCL, PGA and PLA are used for craniomaxillofacial fixation, sutures, interference screws, fixation plates and pins and for meniscal repair. However, the degradation rates of these polymers are not well controlled; they lack mechanical strength and induce inflammation reactions in the acidic medium. To date, few metallic polymer composites such as Mg/PCL have demonstrated compressive moduli similar to human cancellous bone, in addition to their excellent cytocompatibility and osteoblastic differentiation properties. In the current state-of-the-art, biodegradable/bioresorbable composites still require much fundamental research and development for use in bioimplants. Metals have to be selected carefully to avoid metal-related toxicity in the body and to avoid corrosion in the body. There are many other factors to be considered such as the influence of the pH change on adjacent tissue and the metabolic activity in supplementary phases. In the present scenario, magnesium-based implants have good future prospects for development.

## Figures and Tables

**Table 1:** Scaffold characteristics and requirements.

Scaffold Characteristics	Requirements
Biocompatibility Biodegradation/bioresorbability	Non-toxicity to the host tissue, support normal cellular activity, osteoconductive, osteoinductive and osteogenic, angiogenesis A controlled resorption rate to host new bone tissue. Possibility to vary the degradation rate, controlled rate of drug delivery and incorporate biomolecules
Pore size and shape	Minimum pore size of 100 µm for diffusion of nutrients, cell survival and proliferation. To enable bone tissue in-growth, pore sizes in the range of 200–350 µm are required. Multiscale porosity with a combination of micro and macro pores allows cell growth, but may be detrimental in terms of mechanical strength
Mechanical properties	Capability to withstand mechanical stress and loading. Scaffold possibility to have good mechanical properties and to mimic as a natural body component

**Table 2:** Influencing parameters of biodegradation.

Scaffold	Fabrication methodology
	Size/shape
	Pore size and porosity
	surface roughness
	surface area to volume ratio
	Additives or impurities
In vitro factors	pH/ionic strength
	cell type and density
	Mechanical loads
	Incubation temperature
	Biological medium’s composition
In vivo factors	Tissue modelling and remodeling
	Mechanical loads
	Enzyme concentrations
	Implantation site

**Table 3:** Synthetic degradable polymers and applications.

Synthetic Degradable Polymers	Applications
Polycyanoacrylates [61]	Adhesives, drug delivery
Polyanhydrides [62]	Drug delivery
Poly(amino acids) [63]	Drug delivery, tissue engineering, orthopedic applications
Poly(ortho ester) [64]	Drug delivery, Stents
Polyphosphazenes [65]	Blood contacting devices, drug delivery, skeletal reconstruction
Poly(propylene fumarate) [66]	Orthopedic applications
Polylactic acid (PLA) [67], poly glycolic (PGA) [68] and copolymers	Barrier membranes, drug delivery, guided tissue regeneration (in dental applications), orthopedic applications, stents, staples, sutures, tissue engineering
Polyhydroxybutyrate (PHB) [69], polyhydroxyvalerate (PHV) [70], and copolymers	Long-term drug delivery, orthopedic applications, stapes stents
Polycaprolactone [71]	Long-term drug delivery, orthopedic applications, staples, stents
Polydioxanone [72]	Fracture fixation in non-load-bearing bones, sutures, wound clip
